# Relationship Between Hepatitis C Infection and Treatment Status and Coronavirus Disease 2019–Related Hospitalizations in Georgia

**DOI:** 10.1093/infdis/jiae103

**Published:** 2024-03-01

**Authors:** Ana Aslanikashvili, Charlotta Rylander, Tinatin Manjavidze, Amiran Gamkrelidze, Davit Baliashvili, Erik Eik Anda

**Affiliations:** Department of Community Medicine, University of Tromsø—The Arctic University of Norway, Tromsø, Norway; Department of Community Medicine, University of Tromsø—The Arctic University of Norway, Tromsø, Norway; Department of Community Medicine, University of Tromsø—The Arctic University of Norway, Tromsø, Norway; Department of Medical Statistics, National Center for Disease Control and Public Health Georgia, Tbilisi, Georgia; School of Health Sciences, University of Georgia, Tbilisi, Georgia; Task Force for Global Health, Tbilisi, Georgia; Department of Community Medicine, University of Tromsø—The Arctic University of Norway, Tromsø, Norway

**Keywords:** hepatitis C virus, direct-acting antivirals, COVID-19, registry study, Georgia

## Abstract

**Background:**

The aim of this study was to evaluate the impact of hepatitis C virus (HCV) infection and treatment status on coronavirus disease 2019 (COVID-19)–related hospitalizations in Georgia.

**Methods:**

We analyzed 2020–2021 Georgian health registry data for COVID-19–positive individuals and categorized the data by HCV infection and treatment status. Logistic regression was used to assess the strengths of the associations.

**Results:**

Treated individuals with HCV had lower odds of COVID-19–related hospitalization compared to anti-HCV-negative individuals, while untreated HCV-viremic and anti-HCV-positive nonviremic individuals had higher odds.

**Conclusions:**

HCV treatment prior to COVID-19 infection was associated with lower odds of COVID-19–related hospitalization, highlighting the benefits of HCV management in the context of the pandemic.

## Teaser

This Georgian population-based registry study indicates that hepatitis C virus (HCV)–positive individuals treated with direct-acting antivirals may have reduced odds of COVID-19–related hospitalization compared with those who remained untreated for HCV or tested HCV negative.

Hepatitis C virus (HCV) infection is a public health concern; there are an estimated 57.8 million cases globally and 1.5 million new infections per year. Left untreated, HCV can lead to end-stage liver disease, causing an estimated 287 700 deaths annually [1]. The introduction of direct-acting antivirals (DAAs) revolutionized HCV treatment, making >95% of chronic infections curable. DAA treatment regimens are short (up to 12 weeks), well-tolerated, and have minor adverse effects [2].

Severe acute respiratory syndrome coronavirus 2 (SARS-CoV-2) is the cause of coronavirus disease 2019 (COVID-19), for which a global pandemic was declared on 11 March 2020 [3]. As of 31 January 2023, 6.8 million COVID-19–related deaths have been recorded [4].

Lack of specific COVID-19 treatment fueled attempts to repurpose existing, approved antiviral treatments, including DAAs, which have demonstrated potential protective effects against SARS-CoV-2 in in silico and in vivo studies [5].

Georgia (in the Caucasus region), population 3.7 million [6], has a historically high burden of HCV infection. Based on a 2015 nationwide (sero)prevalence survey in adults, the prevalence of active HCV infection was 5.4% and the seroprevalence 7.7% [7]. Georgia introduced nationwide DAA treatment for HCV infection in 2015, the Georgian HCV elimination program. Thanks to Georgia's health registries, we have a unique opportunity to study the relationship between HCV infection and treatment status in relation to COVID-19–related hospitalization in a population setting. Hence, this study aimed to assess whether there were differences in COVID-19–related hospitalization among COVID-19–positive individuals without HCV and those with different HCV infection and treatment status in Georgia.

## METHODS

### Data Sources

This study used data from 5 national Georgian electronic health registries: the HCV Screening Registry, the HCV Elimination Database, the COVID-19 Testing Module, the Hospitalization Registry, and the Immunization Module. Data were merged using personal identification numbers.

We extracted information on anti-HCV and viremia test dates, test results, and DAA treatment dates from the HCV Screening Registry and HCV Elimination Database (time frame: 1 April 2015 [ie, earliest available date] to 31 December 2021). We extracted information on COVID-19 test date, testing method, test result, and demographic data (sex, date of birth, region of residence) from the COVID-19 Testing Module (time frame: 9 September 2020 [ie, the date when COVID-19–related hospitalization criteria changed from universal admission of all positive cases to admission of only severe cases] to 31 December 2021). We collected information on hospitalization (stay at inpatient department for 24 hours or longer) dates and diagnoses from the Hospitalization Registry (time frame: 9 September 2020 to 28 February 2022, to consider the potential delay between COVID-19 diagnosis and related hospitalization).

### Study Sample

**Figure 1.**
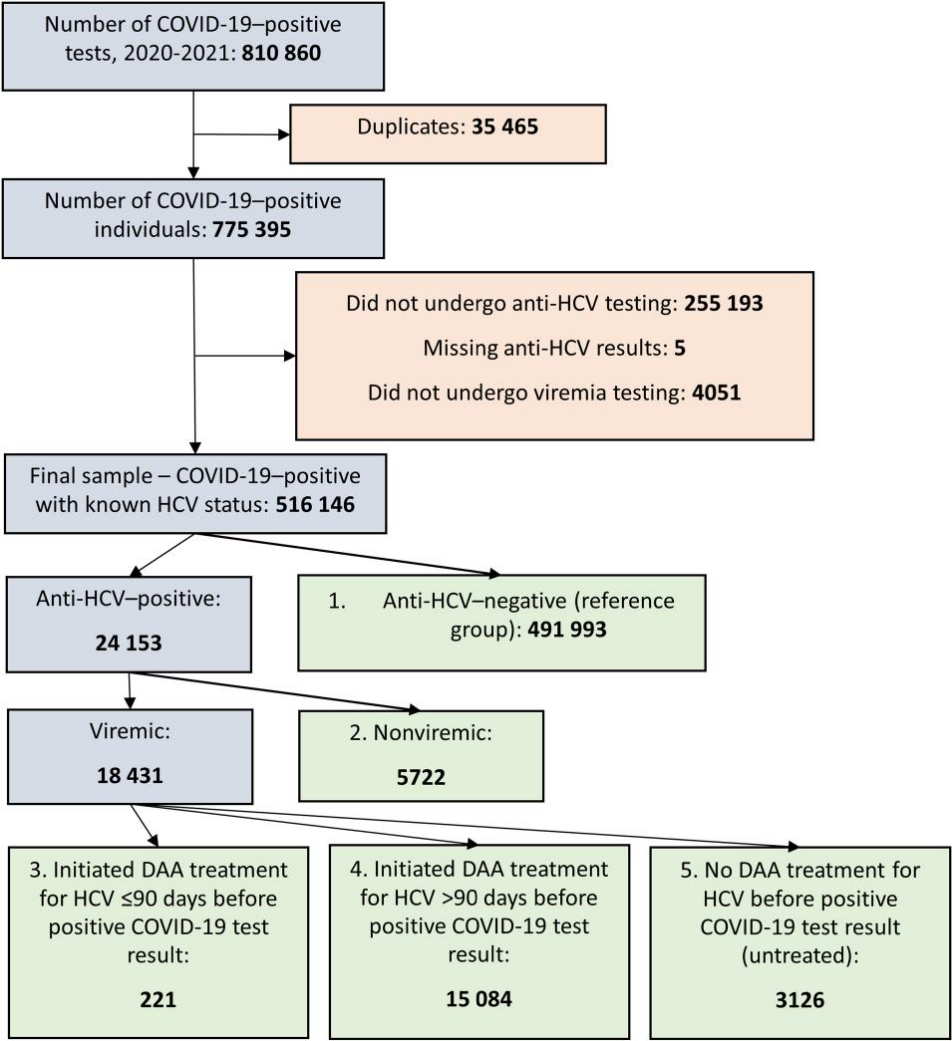
Flowchart of the study sample, showing inclusions (gray), exclusions (peach), and exposure (green) groups. “Viremic” indicates hepatitis C virus RNA/core antigen positive. Abbreviations: COVID-19, coronavirus disease 2019; DAA, direct-acting antiviral; HCV, hepatitis C virus.

This cohort study included all adults (≥18 years of age) with a positive COVID-19 test (by RNA polymerase chain reaction [PCR] or rapid antigen test) recorded in the COVID-19 Testing Module between 9 September 2020 and 31 December 2021, and known HCV infection status. Figure 1 outlines sample selection.

### Definition of Study Groups and Outcomes

According to the Georgian HCV testing protocol, initial screening for HCV is performed with an anti-HCV test. If positive, the person is referred to viremia testing (ie, RNA PCR or “core” antigen chemiluminescent microparticle immunoassay). Viremic cases initiate DAA treatment for HCV with the relevant sofosbuvir-based regimen. We classified participants into 5 exposure groups based on their history of HCV infection and DAA treatment: (1) anti-HCV-negative individuals (ie, reference group, n = 492 001); (2) anti-HCV-positive, nonviremic individuals (n = 5722); (3) viremic individuals who initiated DAA treatment for HCV ≤90 days before positive COVID-19 test result (n = 221); (4) viremic individuals who initiated DAA treatment for HCV >90 days before positive COVID-19 test result (n = 15 084); and (5) viremic individuals with no treatment for HCV before positive COVID-19 test result (ie, untreated, n = 3126) (Figure 1). We selected the period “90 days before a positive COVID-19 test result” because it was the longest DAA regimen available for first-time HCV treatment in Georgia [8].

We defined COVID-19–related hospitalization as registered in the Hospitalization Registry with COVID-19 as the main diagnosis (*International Classification of Diseases, Tenth Revision* codes U07.1 [COVID-19, virus identified], U07.2 [COVID-19, virus not identified], and B34.2 [coronavirus infection, unspecified site]).

### Statistical Analysis

We compared characteristics between the 5 exposure groups. Descriptives were presented as frequencies and percentages. We estimated crude odds ratios (ORs) and adjusted ORs (aORs) of COVID-19 hospitalization using logistic regression models. We used a directed acyclic graph to identify confounders (Supplementary Figure 1) and subsequently adjusted our model for sex and age. Since we excluded individuals who did not undergo anti-HCV testing, we also compared sex and age distributions among COVID-19–positive individuals with and without anti-HCV test results. Additionally, we performed a subanalysis comparing treated individuals to untreated (Supplementary Table 1). The Stata statistical package (StataCorp, College Station, Texas) version 17.0 was used for analysis.

## RESULTS

The anti-HCV-negative reference group consisted of 39.3% men. In contrast, the groups with anti-HCV-positive nonviremic individuals, those who initiated DAA treatment for HCV ≤90 days before COVID-19, and those who started treatment >90 days before COVID-19 comprised 58.3%, 64.7%, and 70.8% men, respectively (Table 1). The reference group was younger than the HCV-positive groups: 40.9% were aged 18–39 years, while viremic individuals who initiated DAA treatment for HCV before COVID-19 were predominantly aged 40–59 years (50.7% and 59.8% of those who initiated DAA treatment ≤90 days and >90 days before COVID-19, respectively). There were no substantial differences in age and sex distributions between COVID-19–positive individuals who had anti-HCV test results and those excluded, but the excluded had a slightly larger proportion of males compared to individuals with anti-HCV test results. The group with available anti-HCV test results had a smaller proportion of individuals aged 18–29 years and a larger proportion of individuals >60 years of age. The proportion of fully vaccinated individuals varied from 7.1% in the untreated group to 13.8% among viremic individuals who initiated DAA treatment for HCV >90 days before COVID-19. The highest proportion of COVID-19–related hospitalizations (36.9%) was observed in the untreated group. COVID-19–related hospitalizations occurred in 18.6% of viremic individuals who initiated DAA treatment for HCV ≤90 days before COVID-19 and 21.6% of those who started treatment >90 days before COVID-19, compared to 24.7% of the reference group (Table 2).

**Table 1.** Characteristics of Individuals With Coronavirus Disease 2019 by Hepatitis C Virus Exposure Group

Characteristic	Anti-HCV-Negative, Reference Group	Anti-HCV-Positive, Nonviremic	Viremic and Initiated DAA Treatment for HCV ≤90 d Before COVID-19	Viremic and Initiated DAA Treatment for HCV >90 d Before COVID-19	Viremic and No DAA Treatment for HCV Before COVID-19	Total
Sex						
Female	298 854 (60.7)	2384 (41.7)	78 (35.3)	4408 (29.2)	1158 (37.0)	306 882 (59.5)
Male	193 139 (39.3)	3338 (58.3)	143 (64.7)	10 676 (70.8)	1968 (63.0)	209 264 (40.5)
Age group, y						
18–29	92 760 (18.9)	316 (5.5)	13 (5.9)	385 (2.6)	72 (2.3)	93 546 (18.1)
30–39	108 028 (22.0)	843 (14.7)	41 (18.6)	2210 (14.7)	419 (13.4)	111 541 (21.6)
40–49	80 597 (16.4)	1388 (24.3)	57 (25.8)	4640 (30.8)	670 (21.4)	87 352 (16.9)
50–59	76 200 (15.5)	1330 (23.2)	55 (24.9)	4370 (29.0)	766 (24.5)	82 721 (16.0)
60–69	74 547 (15.1)	1060 (18.5)	37 (16.7)	2487 (16.5)	571 (18.3)	78 702 (15.3)
70–79	39 580 (8.0)	520 (9.1)	13 (5.9)	799 (5.3)	345 (11.0)	41 257 (8.0)
≥80	20 281 (4.1)	265 (4.6)	5 (2.3)	193 (1.3)	283 (9.1)	21.027 (4.1)
Fully vaccinated^a^	46 253 (9.4)	661 (11.6)	17 (7.7)	2083 (13.8)	222 (7.1)	49 236 (9.5)
Total	491 993	5722	221	15 084	3126	516 146

Data are presented as No. (%). “Viremic” indicates HCV RNA/core antigen positive.

Abbreviations: COVID-19, coronavirus disease 2019; DAA, direct-acting antiviral; HCV, hepatitis C virus.

^a^Received 2 doses ≥14 d before positive COVID-19 test result.

**Table 2.** Odds Ratios and 95% Confidence Intervals for the Association Between the Exposure Groups and Coronavirus Disease 2019–Related Hospitalization

Hospitalizations	Anti-HCV-Negative, Reference Group	Anti-HCV-Positive, Nonviremic	Viremic and Initiated DAA Treatment for HCV ≤90 d Before COVID-19	Viremic and Initiated DAA Treatment for HCV >90 d Before COVID-19	Viremic and No DAA Treatment for HCV Before COVID-19	Total
No. of hospitalizations/No. of total (% hospitalizations)	121 519/491 993 (24.7)	1729/5722 (30.2)	41/221 (18.6)	3256/15 084 (21.6)	1154/3126 (36.9)	127 699/516 147 (24.7)
Crude OR (95% CI)	1.00	1.32 (1.25–1.40)	0.69 (.49–.97)	0.84 (.81–.87)	1.78 (1.66–1.92)	…
Adjusted^a^ OR (95% CI)	1.00	1.15 (1.08–1.22)	0.66 (.46–.93)	0.81 (.77–.84)	1.39 (1.28–1.50)	…

“Viremic” indicates HCV RNA/core antigen positive.

Abbreviations: CI, confidence interval; COVID-19, coronavirus disease 2019; DAA, direct-acting antiviral; HCV, hepatitis C virus; OR, odds ratio.

^a^Adjusted for age and sex.

Compared to anti-HCV-negative individuals with COVID-19, both groups of viremic individuals who initiated DAA treatment for HCV before COVID-19 had lower odds of COVID-19–related hospitalization (≤90 days before: aOR, 0.66 [95% confidence interval {CI}, .46–.93]; >90 days before: aOR, 0.81 [95% CI, .77–.84]). In contrast, the untreated group had increased odds of COVID-19–related hospitalization (aOR, 1.39 [95% CI, 1.28–1.50]); so had the anti-HCV-positive nonviremic individuals (aOR, 1.15 [95% CI, 1.08–1.22]) compared to the reference group (Table 2).

Viremic individuals who initiated DAA treatment for HCV before COVID-19 had considerably lower odds of COVID-19–related hospitalization (≤90 days before: aOR, 0.46 [95% CI, .32–.65]; >90 days before: aOR, 0.57 [95% CI, .52–.62]) compared to viremic and untreated individuals with COVID-19 (Supplementary Table 1).

## DISCUSSION

This is the first national registry-based study to compare differences in COVID-19–related hospitalizations among groups of individuals with different HCV infection and treatment statuses. Our results suggest that individuals who initiated DAA treatment for HCV ≤90 days before COVID-19 had lower odds of COVID-19–related hospitalization (aOR, 0.66 [95% CI, .46–.93]) compared to anti-HCV-negative individuals. Viremic individuals who initiated DAA treatment >90 days before COVID-19 also had lowered odds of COVID-19–related hospitalization (aOR, 0.81 [95% CI, .77–.84]) compared to the reference group. Despite their short half-life, DAAs might have indirect long-term health effects. By eradicating HCV, DAAs enhance general health [9, 10]. Our findings suggest that DAAs may have a protective effect on COVID-19 by hindering progression of the disease to an advanced stage. However, it is possible that people who were currently using or had recently received DAA treatment for HCV may have been more conscious of health and lifestyle habits, in turn contributing to improved COVID-19 outcomes. Although the findings do not entirely preclude the potential for a direct effect of DAAs on SARS-CoV-2, it seems unlikely that this is the predominant factor responsible for the observed results, given that reduced odds of COVID-19–related hospitalizations were observed in groups with both recent and past DAA treatments. The results indicate that there is no reason for individuals to discontinue DAA treatment for HCV during a SARS pandemic.

In contrast to the lower odds of COVID-19–related hospitalization observed among participants who received DAA treatment for HCV, the odds of hospitalization were higher in the untreated group (aOR, 1.39 [95% CI, 1.28–1.50]) when compared to our reference group, presumably because the health status of untreated individuals was aggravated by HCV infection and its complications. For example, COVID-19 can cause liver damage, while the presence of HCV may exacerbate this damage [11], and preexisting liver disease has been linked to worse COVID-19 outcomes [12].

Despite universal access to screening and treatment services for both HCV and COVID-19 in Georgia, individuals who remained untreated may have had characteristics, such as comorbidities or behavioral preferences, which could contribute to increased risk of COVID-19 infection and hospitalization.

Anti-HCV-positive nonviremic individuals had higher odds of COVID-19–related hospitalization (aOR, 1.15 [95% CI, 1.08–1.22]). Because this heterogeneous group includes individuals who had spontaneous clearance of HCV, were previously treated and cured previously, or had false-positive anti-HCV test results, speculation is inadvisable.

The unequal distribution of the study sample by sex and age resembles that in HCV-positive individuals in the Georgian population. Historically, the main risk factors for HCV were injection drug use and a history of blood transfusion [13, 14], linked to the deterioration of social conditions and the collapse of state institutions following the early 1990s civil war. Injection drug use was most prevalent in men, resulting in spread of HCV among males. Women tend to seek HCV diagnostic services more than men within the Georgian HCV elimination program [15], perhaps because of specific health-related behavior among females. The same may apply to young adults, characterized by higher uptake of HCV screening services due to better health awareness and access to digital information channels.

### Strengths and Limitations

In Georgia it is mandatory to report HCV and COVID-19 testing/treatment information to the relevant registries, a process monitored by government institutions. To ensure data completeness and validity, we cross-validated the data based on equal variables, such as sex, age, and region of residence, across different registries.

A limitation is the potential for differences between composition of exposure groups. Varying characteristics among individuals based on initiation of HCV treatment, or how these groups differ from the general population, could have influenced our outcomes and overestimated ORs in both the treated and untreated groups. Those who received HCV treatment may exhibit better health-seeking behavior and more exposure to medical care, which could extend to their responses to COVID-19. The slight differences in age and sex distribution between the included sample and those excluded due to the absence of an anti-HCV test result suggest that there could be variations in the risk of severe COVID-19 between the included and excluded cases.

The rate of COVID-19–related hospitalization varied by COVID-19 variants, with the wave of the Omicron strain being characterized by the lowest rate of severe cases. Our study includes few cases of the Omicron strain, since the first Omicron case was identified in Georgia in mid-December 2021.

## CONCLUSIONS

Our results suggest that there are significant differences in COVID-19–related hospitalizations among groups distinguished by HCV infection and treatment status. Treatment with DAAs appears to be associated with reduced number of COVID-19–related hospitalizations, whereas untreated individuals exhibit a higher number. Ultimately, these results can be interpreted in the direction that there is no reason to avoid DAA treatment for HCV during a SARS pandemic.

## Supplementary Data

Supplementary materials are available at *The Journal of Infectious Diseases* online (http://jid.oxfordjournals.org/). Supplementary materials consist of data provided by the author that are published to benefit the reader. The posted materials are not copyedited. The contents of all supplementary data are the sole responsibility of the authors. Questions or messages regarding errors should be addressed to the author.

## Supplementary Material

jiae103_Supplementary_Data

## Notes

***Acknowledgments.*** Our deepest gratitude goes to all who provided data for this work, including Shaun Shadaker, Vladimer Getia, and Levan Kandelaki, and to Trudy Perdrix-Thoma who contributed with language editing.

***Author contributions.*** Conceptualization: A. A., C. R., A. G., T. M., and E. E. A. Data curation: A. A., T. M., C. R., and E. E. A. Formal analysis and investigation: A. A. Methodology: A. A., C. R., T. M., and E. E. A. Resources and software: A. A. Validation: A. A., C. R., T. M., D. B., A. G., and E. E. A. Visualization: A. A. and E. E. A. Writing—original draft: A. A. Writing—review and editing: C. R., T. M., A. G., D. B., and E. E. A.

***Ethical approval.*** This work was approved by the Institutional Review Board at the National Center for Disease Control and Public Health of Georgia (IRB # 2023–016, 10 February 2023) and the Regional Committees for Medical and Healthcare Research Ethics in Norway (REK) (reference number 577218, 20 February 2023).

***Financial support.*** This work was supported by the University of Tromsø—The Arctic University of Norway (grant number 31122200-215262100). The same institution also provided funding to cover the Open Access publication charges for this article.
