# Supplementary material for: Relationship Between Hepatitis C Infection and Treatment Status and Coronavirus Disease 2019–Related Hospitalizations in Georgia
Source: J Infect Dis. 2024 Mar 1;230(3):e694–9. doi: 10.1093/infdis/jiae103 (PMC11420765; doi:10.1093/infdis/jiae103)
Supplement: jiae103_Supplementary_Data [file jiae103_supplementary_data.zip › Supplementary1.docx]

**Supplementary materials**

**Study setting**

Georgia, an upper-middle-income country in the Caucasus with a population of 3.7 million [1], has a historically high burden of HCV infection. Based on a 2015 nationwide (sero)prevalence survey, the prevalence of active HCV infection in the adult population was 5.4%, and the seroprevalence was 7.7% [2]. Georgia introduced DAA treatment for HCV infection in April 2015, within the framework of the Georgian HCV elimination program. During the study period, available DAA regimens included sofosbuvir, in combination with ledipasvir or velpatasvir, with or without ribavirin. The Georgian HCV elimination program provides HCV-related services free-of-charge and ensures high accessibility. This includes DAA treatment for all individuals with chronic HCV infection, in accordance with international guidelines, regardless of the presence or severity of liver damage. Treatment coverage is high, and the rate of sustained virologic response is 99%. HCV antibody (anti-HCV) testing in Georgia is highly accessible at outpatient facilities and is also routinely performed in certain subgroups of the population, such as pregnant women, hospitalized patients, blood donors, medical and public health personnel, military recruits, and prisoners [3]. At the end of 2021, the Georgian HCV elimination program had tested 2.2 million adults; 76,644 viremic individuals received DAA treatment for HCV (60% of the country target); and the national prevalence of active HCV infection had decreased to 1.8% (67% reduction) [4, 5].

SARS-CoV-2 testing in Georgia has been highly accessible and free-of-charge for symptomatic patients and contacts. By the end of 2021, the country had performed over 13 million tests (RNA polymerase chain reaction [PCR] and rapid antigen tests). Between the detection of the first case of COVID-19 in Georgia on February 26^th^, 2020, and the end of 2021, 936,844 cases had been officially registered, 75.6% of which were reported in 2021. The cumulative COVID-19 fatality rate was 1.48% in the same period. Initially, the national COVID-19 clinical management protocol recommended hospitalization of any confirmed case; however, following the sharp increase in incidence in September 2020, that protocol was changed to recommend hospitalization for severe cases only. COVID-19 vaccination was launched on March 15^th^, 2021, but vaccination uptake has been persistently low. As of January 2022, only 42.3% of the adult population had been fully vaccinated [6].

**Description of study sample**

Of the 810,860 positive test results in the COVID-19 Testing Module, 35,465 duplicates were removed, as we included only a single and the earliest test for all individuals (in cases where multiple tests were recorded). This left 775,395 individuals in the initial sample. After checking the HCV status of these individuals in the HCV Screening Registry and HCV Elimination Database, 255,193 individuals who did not undergo anti-HCV testing and five individuals with missing anti-HCV results were excluded. We also excluded 4,051 individuals who did not receive viremia test, since this group contained an unknown mix of false positive, seropositive, and viremic cases. The final study sample consisted of 516,146 individuals (Figure 1).

**References:**

1. Demographic Situation in Georgia 2021. Tbilisi: National Statistics Office of Georgia; 2022.

2. Hagan LM, Kasradze A, Salyer SJ, Gamkrelidze A, Alkhazashvili M, Chanturia G, et al. Hepatitis C prevalence and risk factors in Georgia, 2015: setting a baseline for elimination. BMC Public Health. 2019;19(Suppl 3):480.

3. Nasrullah M, Sergeenko D, Gvinjilia L, Gamkrelidze A, Tsertsvadze T, Butsashvili M, et al. The Role of Screening and Treatment in National Progress Toward Hepatitis C Elimination - Georgia, 2015-2016. MMWR Morb Mortal Wkly Rep. 2017;66(29):773-6.

4. Gamkrelidze A, Shadaker S, Tsereteli M, Alkhazashvili M, Chitadze N, Tskhomelidze I, et al. Nationwide hepatitis C serosurvey and progress towards HCV elimination in the country of Georgia, 2021. J Infect Dis. 2023.

5. Strategic Plan for the Elimination of Hepatitis in Georgia 2021-2025. 2021.

6. COVID-19 Report of the National Center for Disease Control and Public Health 2020-2021. Tbilisi: National Center for Disease Control and Public Health Georgia; 2022.
