# Supplementary material for: Relationship Between Hepatitis C Infection and Treatment Status and Coronavirus Disease 2019–Related Hospitalizations in Georgia
Source: J Infect Dis. 2024 Mar 1;230(3):e694–9. doi: 10.1093/infdis/jiae103 (PMC11420765; doi:10.1093/infdis/jiae103)
Supplement: jiae103_Supplementary_Data [file jiae103_supplementary_data.zip › Supplementary2_table1.docx]

**Supplementary table 1**. Odds ratios (OR) and 95% confidence intervals (CI) for the association between exposure groups and COVID-19-related hospitalization using strictly HCV-infected individuals.

| Exposure groups | n hospitalizations/ n total (% hospitalizations) | Crude OR (95% CI) | Adjusted^[[1]](#footnote-1)^ OR (95% CI) |
| --- | --- | --- | --- |
| **Viremic and no DAA treatment for HCV before COVID-19 (reference group)** | 1,154/3,126 (36.9) | 1.00 | 1.00 |
| **Viremic and initiated DAA treatment for HCV ≤90 days before COVID-19** | 41/221 (18.6) | 0.39 (0.28-0.55) | 0.46 (0.32-0.65) |
| **Viremic and initiated DAA treatment for HCV >90 days before COVID-19** | 3,256/15,084 (21.6) | 0.47 (0.43-0.51) | 0.57 (0.52-0.62) |

Viremic= HCV RNA/cAg-positive. Abbreviations: OR (Odds ratio), CI (Confidence interval), COVID-19 (Coronavirus disease 2019), HCV (Hepatitis C virus), DAA (Direct acting antiviral).

1. Adjusted for age and sex. [↑](#footnote-ref-1)
