# Supplementary material for: Relationship Between Hepatitis C Infection and Treatment Status and Coronavirus Disease 2019–Related Hospitalizations in Georgia
Source: J Infect Dis. 2024 Mar 1;230(3):e694–9. doi: 10.1093/infdis/jiae103 (PMC11420765; doi:10.1093/infdis/jiae103)
Supplement: jiae103_Supplementary_Data [file jiae103_supplementary_data.zip › Supplementary3_figure1.docx]

**Supplementary figure 1.** Directed acyclic graph.


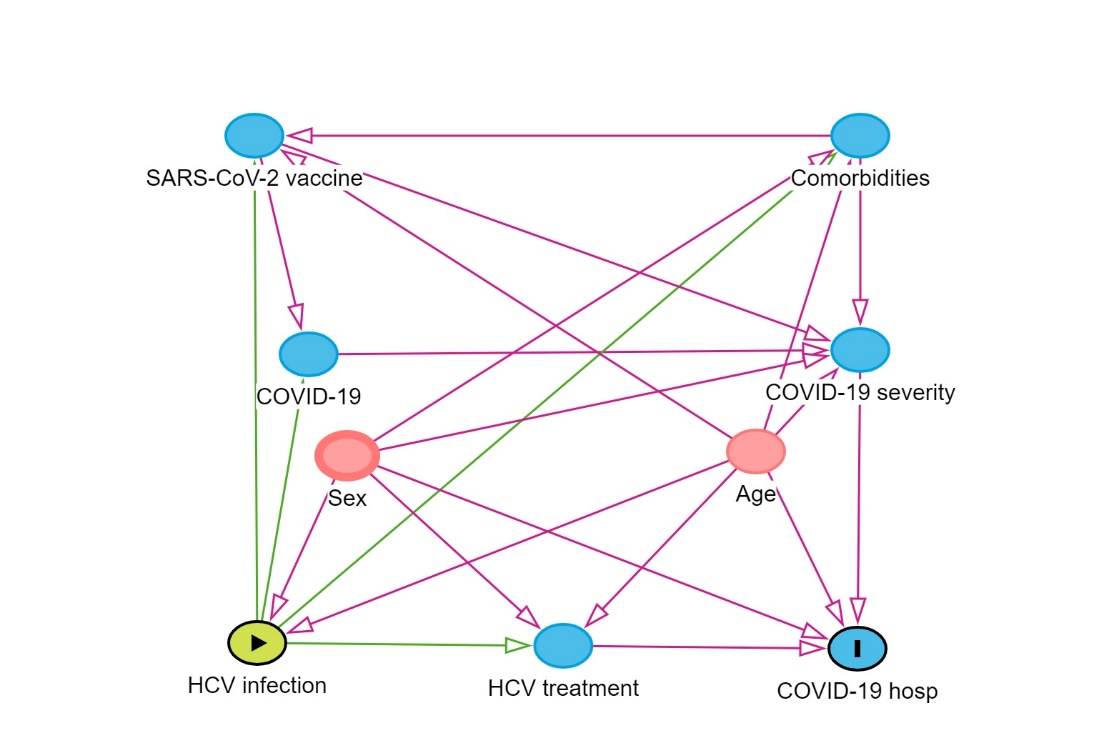


We constructed directed acyclic graph (DAG) to explain the association between hepatitis C virus (HCV) infection/treatment and COVID-19-related hospitalizations using the DAGgity online tool [1].

It is hypothesized that individuals with known HCV status are more conscious of their health and are thus more likely to utilize preventive medical services, such as the SARS-CoV-2 vaccine. Those who exhibit clinical manifestation of HCV or who are undergoing HCV treatment may be at an increased risk of contracting COVID-19 due to more frequent visits to healthcare facilities.

HCV is associated with extrahepatic manifestations, including cryoglobulinemia, atherosclerosis, and metabolic disorders, which can precipitate the development of various diseases [2, 3, 4]. These comorbidities can exacerbate the severity of COVID-19 and elevate the risk of COVID-19-related hospitalization.

HCV infection and treatment, as well as COVID-19-related hospitalization are impacted by age and sex. Consequently, we adjusted for these variables, considering them a minimal sufficient adjustment set, to estimate the association between HCV and COVID-19-related hospitalization.

References:

1. Textor J, van der Zander B, Gilthorpe MS, Liskiewicz M, Ellison GT. Robust causal inference using directed acyclic graphs: the R package 'dagitty'. Int J Epidemiol. 2016;45(6):1887-94.

2. Mazzaro C, Quartuccio L, Adinolfi LE, Roccatello D, Pozzato G, Nevola R, et al. A Review on Extrahepatic Manifestations of Chronic Hepatitis C Virus Infection and the Impact of Direct-Acting Antiviral Therapy. Viruses. 2021;13(11).

3. Rosenthal E, Cacoub P. Extrahepatic manifestations in chronic hepatitis C virus carriers. Lupus. 2015;24(4-5):469-82.

4. Adinolfi LE, Rinaldi L, Nevola R. Chronic hepatitis C, atherosclerosis and cardiovascular disease: What impact of direct-acting antiviral treatments? World J Gastroenterol. 2018;24(41):4617-21.
